# Clustering of patients with overactive bladder syndrome

**DOI:** 10.1186/s12894-021-00812-9

**Published:** 2021-03-19

**Authors:** James Gross, Joel M. Vetter, H. Henry Lai

**Affiliations:** 1 Division of Urologic Surgery, Department of Surgery, Washington University School of Medicine, 4960 Children’s Place, Campus, Box 8242, St Louis, MO 63110 USA; 2 Department of Anesthesiology, Washington University School of Medicine, St Louis, MO USA

**Keywords:** Overactive bladder, Clustering, Phenotyping

## Abstract

**Background:**

Overactive bladder is a heterogenous condition with poorly characterized clinical phenotypes. To discover potential patient subtypes in patients with overactive bladder (OAB), we used consensus clustering of their urinary symptoms and other non-urologic factors.

**Methods:**

Clinical variables included in the k-means consensus clustering included OAB symptoms, urinary incontinence, anxiety, depression, psychological stress, somatic symptom burden, reported childhood traumatic exposure, and bladder pain.

**Results:**

48 OAB patients seeking care of their symptoms were included. k-means consensus clustering identified two clusters of OAB patients: a urinary cluster and a systemic cluster. The systemic cluster, which consisted of about half of the cohort (48%), was characterized by significantly higher psychosocial burden of anxiety (HADS-A, 9.5 vs. 3.7, p < 0.001), depression (HADS-D, 6.9 vs. 3.6, p < 0.001), psychological stress (PSS, 21.4 vs. 12.9, p < 0.001), somatic symptom burden (PSPS-Q, 28.0 vs. 7.5, p < 0.001), and reported exposure to traumatic stress as a child (CTES, 17.0 vs. 5.4, p < 0.001), compared to the urinary cluster. The systemic cluster also reported more intense bladder pain (3.3 vs. 0.8, p = 0.002), more widespread distribution of pain (34.8% vs. 4.0%, p = 0.009). The systemic cluster had worse urinary incontinence (ICIQ-UI, 14.0 vs. 10.7, p = 0.028) and quality of life (SF-36, 43.7 vs. 74.6, p < 0.001). The two clusters were indistinguishable by their urgency symptoms (ICIQ-OAB, OAB-q, IUSS, 0–10 ratings). The two OAB clusters were different from patients with IC/BPS (worse urgency incontinence and less pain).

**Conclusions:**

The OAB population is heterogeneous and symptom-based clustering has identified two clusters of OAB patients (a systemic cluster vs. a bladder cluster). Understanding the pathophysiology of OAB subtypes may facilitate treatments.

## Background

Overactive bladder (OAB) affects 1 in 6 adults in the United States and has an economic cost of $24.9 billion [1, 2]. Despite the enormous burden and negative impact on quality of life, our understanding of the underlying pathophysiology is poor. Treatment outcomes remain suboptimal overall. Most patients do not continue their medications one year after the prescription [3], and many patients are “refractory” to OAB treatments.

Recent research suggested that non-urologic factors such as anxiety, depression, psychological stress, somatic symptom burden, non-urologic pain, and increased hypersensitivity related to central sensitization might contribute to the symptomatology of OAB and other lower urinary tract symptoms (LUTS) [4–10]. This observation raises the possibility that the OAB population is heterogeneous and may be further sub-categorized based on their non-urologic factors in addition to their urinary symptoms.

Consensus clustering provides a means to discover patient subtypes in patient population with heterogeneous presentation. Unlike traditional comparative approach, clustering algorithms use empirical statistical methods to discover subtypes based on intrinsic patterns within the data without making any a priori assumption or preconceived notion on how the classification scheme should be constructed. This unbiased, data driven approach may provide novel insights and more precise classification of patient subtypes. To our knowledge, consensus clustering has not been reported for OAB patients. In this study, we incorporated patients’ urinary symptoms and non-urologic factors into consensus clustering to identify potential patient subtypes within OAB.

## Materials and methods

### OAB participants

Male and female patients with OAB seen between October 2012 and July 2014 were approached to participate in this research study. The case definition of OAB was consistent with the 2002 International Continence Society terminology [11]. Specifically, OAB patients must have complaints of urinary urgency, with or without urgency urinary incontinence, usually with frequency and nocturia, in the absence of other causes. Exclusion criteria included: history of prostate surgery, incontinence surgery, urethral stricture, neurogenic bladder, urinary retention, pelvic radiation, cyclophosphamide cystitis, tuberculosis cystitis, urologic cancer, urinary stones, positive urine culture in the past 6 weeks, or residual volume ≥ 150 mL. The study was approved by Washington University Institutional Review Board. All participants signed an informed consent.

### Assessment

Urinary symptoms, psychosocial symptoms, bladder pain, systemic pain, and quality of life were assessed using validated questionnaires.

OAB symptoms were assessed using the International Consultation on Incontinence—Overactive Bladder (ICIQ-OAB) [12] and OAB-q short form [13]. Urgency symptoms were assessed using the Indevus Urgency Severity Scale (IUSS) [14] and on a 0–10 numeric rating scale of urgency. Urinary incontinence symptoms were assessed using International Consultation on Incontinence—Urinary Incontinence short form (ICIQ-UI) [15] and Incontinence Impact Questionnaire Short Form (IIQ-7) [16].

The following psychosocial symptoms were assessed: (1) anxiety (Hospital Anxiety and Depression Scale, HADS) [17], (2) depression (HADS) [17], (3) psychological stress (Perceived Stress Scale, PSS) [18], (4) somatic symptom burden (Poly-Symptomatic, Poly-Syndromic Questionnaire, PSPS-Q) [19], and (5) reported exposure to various childhood trauma (Childhood Traumatic Events Scale, CTES) [20].

Intensity of bladder pain was assessed using a 0–10 numeric rating scale. Intensity of non-urologic pain was assessed using the Brief Pain Inventory (BPI) [21]. The distribution of pain was assessed using a whole body map as previously described [22]. Those reporting pain in 3 or more broader body regions outside the pelvis (left or right upper extremity, left or right lower extremity, head and neck, chest, lower back) were classified as having “widespread pain” [22].

Condition-specific quality of life was assessed using the OAB-q QOL subscale (OAB-q-HRQOL) [13]. Global quality of life was assessed using the SF-36.

### Clustering analysis

Variables entering k-means consensus clustering included urinary incontinence (ICIQ-UI), OAB symptoms (ICIQ-OAB), anxiety (HADS), depression (HADS), psychological stress (PSS), somatic symptom burden (PSPS-Q), reported exposure to childhood trauma (CTES), and bladder pain (0–10). K-means uses Euclidean distance to group participants into clusters, while assigning observations to clusters in order to minimize the distance between observations and the mean or center of the cluster, or the total intra-cluster variation [23, 24]. The optimal number of clusters was estimated using the elbow method and compared to a number of different techniques for determining number of clusters using the *NbClust* package within the R statistical software to validate the choice [25]. We performed one-way ANOVA and chi-square tests for continuous and categorical variables respectively to test for differences between clusters. p < 0.05 was considered significant. All statistical analyses utilized the open source statistical package R v3.3.1.

### Comparing the discovered oab clusters to interstitial cystitis/bladder pain syndrome

Since one of the identified OAB clusters had pain and psychosocial symptoms (see results below), in order to verify that our OAB population was distinct from interstitial cystitis/ bladder pain syndrome (IC/BPS), we compared the bladder pain and urinary incontinence scores of our two identified OAB clusters to an IC/BPS cohort that was previously described [26]. IC/BPS patients were required to have an unpleasant sensation (pain, pressure, discomfort) perceived to be related to the bladder, associated with lower urinary tract symptoms of more than 6 weeks duration, in the absence of infection or other identifiable causes, in accordance with the 2011 AUA Guideline [27]. The distinction between OAB and IC/BPS was based on the two published AUA Guidelines [27, 28], chief complaint, and overall clinical impression, taken into the account of their clinical evaluation and management (e.g., antimuscarinics for OAB, tricyclics for IC/BPS). Additionally, we assessed the likely diagnosis of the OAB patients by applying a previously described independent nomogram, which has a 94% accuracy for classifying or distinguishing patients as likely OAB versus likely IC/BPS based on GUPI, ICSI, and OAB-q [29]. Due to missing data, the Urge Incontinence Composite Index was based on only questions four and eight of the OAB-q.

## Results

**Fig. 1:**
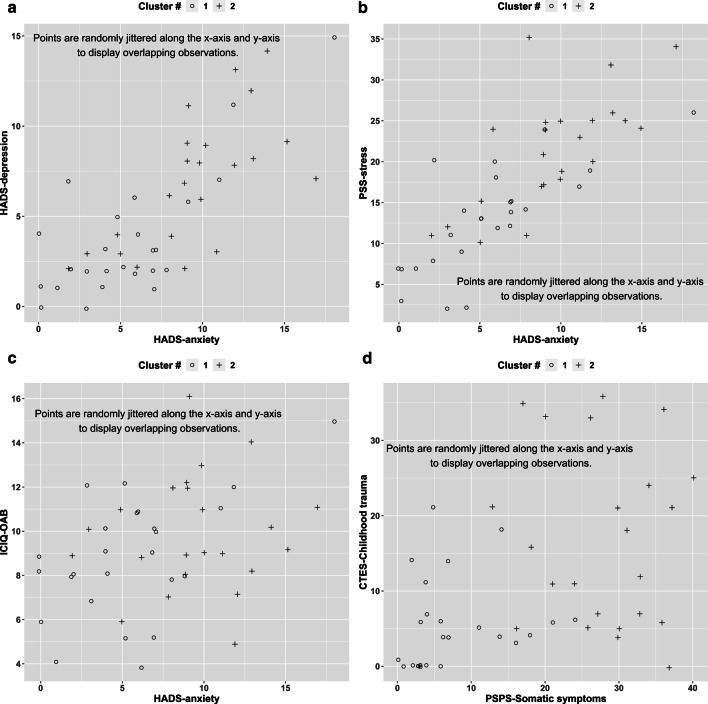
Distribution of urinary and psychosocial measures. o = urinary cluster, +  = systemic cluster

48 OAB patients (13 men, 35 women) had complete data for consensus clustering. Their characteristics are presented in Table 1. k-means clustering identified two OAB clusters: a urinary cluster and systemic cluster. The two clusters are illustrated in selected scatter plots in Fig. 1 (+ = systemic cluster, o = urinary) Comparisons between the two clusters are shown in Table 1.

**Table 1:** Comparisons between the two OAB clusters, mean ± SD

	Urinary cluster (n = 25, 52%)	Systemic cluster (n = 23, 48%)	p-value
Demographics			
Age (mean ± SD)	53.8 ± 13.9	54.2 ± 10.0	0.84
No. of females	16	19	0.20
Urinary symptoms: (mean ± SD)			
Urinary incontinence (ICIQ-UI, 0–21)^	10.7 ± 5.1	14.0 ± 4.1	0.028*
Incontinence impact (IIQ-7, 0–28)	6.5 ± 7.4	11.7 ± 8.6	0.027*
Overactive bladder (ICIQ-OAB, 0–16)^	8.8 ± 2.7	9.9 ± 2.6	0.20
Overactive bladder symptom severity (OAB-q SS, 6–36)	58.5 ± 20.0	69.3 ± 22.4	0.11
Psychosocial			
Anxiety (HADS-A, 0–21)^	5.5 ± 4.1	9.5 ± 3.7	< 0.001*
Depression (HADS-D, 0–21)^	3.7 ± 3.5	6.9 ± 3.6	< 0.001*
Psychological stress (PSS, 0–40)^	12.9 ± 6.4	21.4 ± 7.0	< 0.001*
Somatic symptom burden (PSPS-Q, 0–59)^	7.5 ± 6.6	28.0 ± 7.6	< 0.001*
Childhood traumatic exposure (CTES, 0–42)^	5.4 ± 6.0	17.0 ± 11.6	< 0.001*
Death in family	2.1 ± 2.8	3.7 ± 3.2	0.070
Parental upheaval	0.8 ± 1.8	2.7 ± 3.2	0.021*
Sexual trauma	0.6 ± 1.9	3.1 ± 3.2	0.004*
Victim of violence	0.6 ± 1.9	2.5 ± 3.2	0.017*
Major illness	0.2 ± 0.8	2.4 ± 2.9	0.001*
Other trauma	1.2 ± 2.4	2.6 ± 3.0	0.055
Quality of life			
Condition specific (OAB-q-HRQOL, 0–100, higher is worse)	40.3 ± 22.6	56.0 ± 26.9	0.045*
Global QOL (SF-36, 0–100, lower is worse)	74.6 ± 17.8	43.7 ± 18.4	< 0.001*

*p < 0.05. ^^^ identifies variables that are used in the clustering algorithm

There was a near equal percent split between the two clusters (52% urinary, 48% systemic). There were no age and sex differences between the two clusters.

The systemic cluster was characterized by significantly higher psychosocial burden of anxiety (HADS-A, 9.5 vs. 3.7, p < 0.001), depression (HADS-D, 6.9 vs. 3.6, p < 0.001), psychological stress (PSS, 21.4 vs. 12.9, p < 0.001), somatic symptom burden (PSPS-Q, 28.0 vs. 7.5, p < 0.001), and reported exposure to traumatic stress as a child (CTES, 17.0 vs. 5.4, p < 0.001), compared to the urinary cluster. The systemic cluster also reported more intense bladder pain (3.3 vs. 0.8 on a 0–10 scale, p = 0.002), more intense non-urologic pain (BPI, 3.1 vs. 1.7, p = 0.021), and more widespread distribution of pain (34.8% vs. 4.0%, p = 0.009). The systemic cluster had worse urinary incontinence (ICIQ-UI, 14.0 vs. 10.7, p = 0.028), condition-specific quality of life (OAB-q-HRQOL, 56.0 vs. 40.3, p = 0.045), and global quality of life (SF-36, 43.7 vs. 74.6, p < 0.001). The two clusters were indistinguishable by their urgency symptoms (no differences in ICIQ-OAB, OAB-q, IUSS, and 0–10 urgency ratings, p all > 0.05).

There were strong correlations between anxiety, depression, and stress across the two clusters (r = 0.78–0.62, see Table 2). This is illustrated in Fig. 1A, B. Correlations between psychosocial and urinary measures were weaker (r = 0.42 to 0.09, Fig. 1C). A combination of high somatic symptom burden and high childhood traumatic scores (blue dots in top right corner of Fig. 1D) appeared to separate the systemic cluster from the urinary cluster which has both low somatic symptom and low childhood traumatic scores (red dots in the bottom left corner).

**Table 2:** Correlation coefficients between urinary and psychosocial measures

	HADS-D (depression)	HADS-A	PSS	PSPS-Q	CTES	SHCU Q2	ICIQ-UI
ICIQ-OAB (overactive bladder)	0.30	0.37	0.24	0.17	0.11	− 0.09	0.43
ICIQ-UI (urinary incontinence)	0.39	0.42	0.40	0.40	0.09	0.16	
SHCU Q2 (bladder pain)	0.23	0.18	0.32	0.45	0.39		
CTES (childhood traumatic exposure scale)	0.07	0.16	0.20	0.44			
PSPS-Q (somatic symptom burden)	0.38	0.41	0.54				
PSS (stress)	0.62	0.78					
HADS-A (anxiety)	0.77						

We compared the bladder pain and urinary incontinence scores of our two identified OAB clusters to a known IC/BPS cohort to verify that our OAB population was not an IC/BPS population that was misclassified as patients with OAB. Results showed that the systemic cluster had significantly less bladder pain (3.3 vs. 6.6, p < 0.001) and more urinary incontinence (ICIQ-UI, 14.0 vs. 6.6, p < 0.001) compared to IC/BPS patients (Table 3). Additionally, we found that 46 of the 48 patients within our clinically diagnosed OAB cohort, which includes both the urinary and systemic cluster patients, were classified as likely OAB using the nomogram described by Ackerman et al. [29]. The additional 2 patients had insufficient data to use the nomogram.

**Table 3:** Comparisons of the two identified clusters to IC/BPS

	Urinary cluster (n = 25)	Systemic cluster (n = 23)	IC/BPS comparison group (n = 27)	p-value, urinary OAB versus IC/BPS	p-value, systemic OAB versus IC/BPS
Age (mean ± SD)	53.8 ± 13.9	54.2 ± 10.0	44.8 ± 16.6	0.036*	0.028*
No. of females	16	19	27	< 0.001*	0.038*
Bladder pain (0–10)	0.8 ± 1.7	3.3 ± 3.0	6.6 ± 2.1	< 0.001*	< 0.001*
Urinary incontinence (ICIQ-UI, 0–21)	10.7 ± 5.1	14.0 ± 4.1	6.6 ± 5.1	0.012*	< 0.001*

*p < 0.05

## Discussion

We identified two subtypes of OAB patients using symptom-based consensus clustering: a urinary cluster and a systemic cluster. The systemic cluster, which consisted of about half of the cohort, was characterized by significantly higher psychosocial burden of anxiety, depression, psychological stress, somatic symptom burden, and reported exposure to traumatic stress as a child, compared to the urinary cluster. The systemic cluster also reported more intense bladder pain, more widespread distribution of pain, worse urinary incontinence, and poorer quality of life.

We believe that the systemic cluster was not simply an IC/BPS population mimicking OAB. Even though the systemic cluster had higher pain and psychosocial symptoms compared to the urinary cluster, the systemic cluster had significantly less bladder pain and more urinary incontinence compared to IC/BPS. Furthermore, the patients in both clusters were classified as OAB using a previously described independent nomogram, which had a diagnostic accuracy of 94% in their validation cohort [29]. These findings make it unlikely that patients in the systemic cluster had IC/BPS, which further supports this previously unrecognized cluster as a subtype of OAB.

Many of the differences between the two clusters were not only statistically different, but likely were clinically significant. The anxiety, stress, somatic symptom, and childhood trauma scores in the systemic cluster were 73%, 66%, 270% and 220% higher respectively than those in the urinary cluster. The systemic cluster had bladder pain in the mild pain category (mean 3.3 on a 0–10 scale) vs. minimal pain (mean 0.8) in urinary cluster. The systemic cluster was 7.7 times more likely to report widespread pain.

Even with different questionnaires (ICIQ-OAB, OAB-q, IUSS, and 0–10 urgency ratings), patients in our two identified clusters had indistinguishable urgency symptoms. This indicates that those patients belonging to the group with more systemic difficulties would be hard to distinguish from their counterparts in a setting where patients with OAB are only evaluated in regard to their syndrome defining urinary symptoms. Additional instruments (e.g., HADS and a body map) are needed to distinguish between these two groups in a clinical setting.

The identification of a systemic cluster in OAB is new. It appears that a subset of OAB patients (the systemic cluster) may not be “just” an OAB patient with “only” bladder symptoms. The finding of increased psychosocial burden and non-zero pain in OAB was relatively new in the literature. In a study comparing OAB and IC/BPS patients, 33% of OAB patients reported pain or discomfort associated with bladder filling [26]. A subset of OAB patients also reported urogenital pain and widespread pain [30]. The presence of pelvic pain was associated with worse psychosocial health [30]. High anxiety, depression, psychological stress, and somatic symptom burden were also associated with more severe urinary incontinence, and poorer quality of life [4–7, 10]. Our consensus clustering results have grouped these patients into a distinct systemic cluster. Notice that there were strong correlations among the various psychosocial measures, suggesting that individuals high in one psychosocial construct (e.g., anxiety) were more likely to have other psychosocial measures (e.g., depression, stress, see Fig. 1A, B).

Consideration of non-urologic factors such as psychological and pain profiles might be important in clinical phenotyping of OAB. Our results highlighted that the OAB population is heterogeneous and there may be different subtypes of OAB (a systemic cluster vs. urinary cluster). It is unclear whether the two identified clusters might have different underlying pathophysiology. Conceptually it is possible that the systemic cluster might have “top-down” or systemic mechanisms such as central sensitization, systemic inflammation, or psychosocial trauma. Central sensitization has recently been demonstrated in some OAB patients by Reynolds et al. [8, 9]. In future studies it is important to study the differences in pathophysiology among the OAB subtypes.

At this time, it is unclear what the therapeutic implications are in terms of treatments. We do not know whether there will be differential responses to OAB treatments between the two identified clusters. We hypothesize that the systemic cluster might be less responsive to traditional OAB treatments while those in the urinary cluster might respond more favorably. Theoretically one might consider the systemic cluster to be out of boundary of bladder-centric treatments and therefore might be less responsive or non-responsive to traditional treatments of OAB that focus on the bladder (e.g., oral antimuscarinics, beta-agonist, botox injection, pelvic floor therapy). This systemic cluster may explain why some OAB patients were “refractory” to traditional treatments of OAB. It may also explain why OAB as a whole can be difficult to treat effectively because it is a heterogeneous condition with many subtypes that are poorly understood.

Reynolds et al. have shown that patients requiring third line treatment for OAB (“refractory” patients) demonstrated higher rates of central sensitization when compared to patients who were first presenting for OAB treatment (treatment naïve) [9]. This observation lends credence to our hypothesis. It also supports the validity of this systemic cluster as a subgroup of patients within OAB that can be identified using clinical measures and potentially treated more effectively with different therapies than their counterparts in the urinary cluster. A larger cohort with longer term treatment data is necessary to investigate this hypothesis.

Further research is needed to validate the two identified OAB clusters, and to further assess whether or not the clusters identified here would respond differentially to OAB treatments. At this time we are not ready to advocate the use of additional questionnaires in clinical practice to assess non-urologic features in OAB patients. We need more research to assess the additional value of evaluating OAB patients with respect to their systemic profiles.

The limitations of this study are inherent to the use of clustering algorithms and the subjective nature of patient reported symptoms. The characteristics included in the model will impact how the patients can be grouped optimally. While these limitations exist, previous work supports the presence of an OAB subtype that fits the characteristics of our systemic cluster and suggests the potential for improvement in treatment algorithms if these subtypes could be identified in clinical practice. Another limitation is the small sample size. Future work should focus on establishing the reproducibility of these clusters in a larger data set, establishing the thresholds for membership between these clusters, assessing if there are treatment response differences between them, and examining their differences in pathophysiology. Moving clinical phenotyping research beyond symptom-based classification by incorporating other mechanistic data (e.g., biomarkers, functional MRI) in the future is also important.

## Conclusions

The OAB population is heterogeneous and symptom-based clustering has identified two clusters of OAB patients (a systemic cluster vs. a urinary cluster). Understanding the pathophysiology of OAB subtypes may facilitate treatments.

## Data Availability

Since the study and manuscript development is ongoing, we are not yet ready to release the raw data to an open data repository. Interested researchers should contact the corresponding author Dr. H. Henry Lai directly for discussion for collaboration.

## Abbreviations

OAB: Overactive bladder
IC/BPS: Interstitial cystitis/bladder pain syndrome
ICIQ-UI: International consultation on incontinence—urinary Incontinence short form
IIQ-7: Incontinence impact questionnaire short form
ICIQ-OAB: International consultation on incontinence—overactive bladder
IUSS: Indevus urgency severity scale
HADS: Hospital anxiety and depression scale
PSS: Perceived stress scale
PSPS-Q: Poly-symptomatic, poly-syndromic questionnaire
CTES: Childhood traumatic events scale
BPI: Brief pain inventory
OAB-q-HRQOL: OAB-q QOL subscale
